# A Geometry Effect of Carbon Nanomaterials on Flame Retardancy and Mechanical Properties of Ethylene-Vinyl Acetate/Magnesium Hydroxide Composites

**DOI:** 10.3390/polym10091028

**Published:** 2018-09-14

**Authors:** Zhi-Qi Liu, Zhi Li, Yun-Xian Yang, Yan-Ling Zhang, Xin Wen, Na Li, Can Fu, Rong-Kun Jian, Li-Juan Li, De-Yi Wang

**Affiliations:** 1 Key Laboratory of Comprehensive and Highly Efficient Utilization of Salt Lake Resources, Qinghai Institute of Salt Lakes, Chinese Academy of Sciences, Xining 810008, China; zqliu@isl.ac.cn (Z.-Q.L.); ylzhang@isl.ac.cn (Y.-L.Z.); lina162@mails.ucas.ac.cn (N.L.); lilj@isl.ac.cn (L.-J.L.); 2 Qinghai Engineering and Technology Research Center of Comprehensive Utilization of Salt Lake Resources, Xining 810008, China; 3 IMDEA Materials Institute, C/Eric Kandel, 2, 28906 Getafe, Spain; zhi.li@imdea.org (Z.L.); yunxian.yang@imdea.org (Y.-X.Y.); hgwenxin@126.com (X.W.); can.fu@imdea.org (C.F.); jrkht1987@fjnu.edu.cn (R.-K.J.); 4 Universidad Politécnica de Madrid, E.T.S. de Ingenieros de Caminos, Madrid 28040, Spain; 5 Nanomaterials Physicochemistry Department, Faculty of Chemical Technology and Engineering, West Pomeranian University of Technology in Szczecin, 70-311 Szczecin, Poland; 6 University of Chinese Academy of Sciences, Beijing 100049, China; 7 Fujian Provincial Key Laboratory of Polymer Materials, College of Chemistry and Materials Science, Fujian Normal University, Fuzhou 350007, China

**Keywords:** ethylene-vinyl acetate, magnesium hydroxide, carbon material, flame retardant

## Abstract

This study was aimed at investigating the effects of carbon nanomaterials with different geometries on improving the flame retardancy of magnesium hydroxide–filled ethylene-vinyl acetate (EM). The thermal stability and flame retardancy were studied by thermogravimetric analysis (TGA), limiting oxygen index (LOI), UL-94 test, and cone calorimeter test (CCT). The in situ temperature monitoring system and interrupted combustion offered direct evidence to link flame retardancy and composite structure. Results demonstrated that carbon nanomaterials enhanced the thermal stability and fire safety of EM. The geometry of carbon nanomaterials played a key role in synergistic flame retardancy of EM, with the flame-retardant order of carbon nanotube > nanoscale carbon black > graphene. Based on an online temperature monitoring system and interrupted combustion test, one-dimensional carbon nanotube was more inclined to form the network structure synergistically with magnesium hydroxide in ethylene-vinyl acetate, which facilitated the generation of more continuous char structure during combustion. In parallel, the mechanical property was characterized by a tensile test and dynamic mechanical analysis (DMA). The incorporation of carbon nanomaterials presented a limited effect on the mechanical properties of the EM system.

## 1. Introduction

Ethylene-vinyl acetate (EVA) is a typical thermoplastic elastomer that is extensively applied in hot-melt adhesive, flexible pipe, battery adhesive film or toys, and especially in the cable industry as an excellent insulating material with good physical and mechanical properties [1]. However, EVA can be ignited easily and burns very rapidly, producing enormous heat and toxic gas. Even under an oxygen-deficient environment, combustion is sustained with the generation of melt dripping, which restricts its practical applications [2]. One of the most effective ways to overcome this drawback is to add flame retardants to EVA, and recently much research is focused on the use of environmentally friendly halogen-free flame-retardant (HFFR) additives [3].

As an HFFR filler, magnesium hydroxide (MH) is extensively used to prepare flame-retardant composites, because of its good smoke suppression and high thermal decomposition temperature [4,5]. There have been many reports about MH applications in polymers, such as polyvinyl chloride (PVC) [6], polypropylene (PP) [7], polyethylene (PE) [8], epoxy resin [9], etc. However, it has a serious disadvantage of low flame-retardant efficiency, and it commonly requires more than 50 wt % MH to meet flame-retardancy of polymers, which inevitably results in the deterioration of physical and mechanical properties of polymers. Therefore, various studies have been done to enhance the flame retardancy and decrease the loading of MH by combining MH with other synergistic additives, such as silica [1], red phosphorous [10], hollow glass microsphere [4], zinc borate [11], clay [12,13], layered double hydroxide [14], etc.

To date, carbon nanomaterials with high thermal conductivity, high strength, flexibility, and low density have attracted considerable attention for developing high-performance polymer composites [15,16,17,18,19]. Interestingly, except for the strengthening effect, the incorporation of carbon nanomaterials is also found to improve the flame retardancy of polymers. Dittrich et al. [20] reported that the peak heat release rate (PHRR) of composites with 1% multiwall carbon nanotubes (CNs) was reduced by about 73% compared to that of PP. Wen and Yang et al. [21,22] revealed that nanocarbon black (CB) could increase the thermal stability and flame retardancy of polyolefin due to its trapping radical effect during the burning of composites. Graphene (CG) has attracted tremendous attention and research interest in the scientific community [23,24]. Liu et al. [25] reported that after pristine graphene or graphene oxide was incorporated into thermosetting resins, the PHRR of composites displayed a decreasing trend compared with pure epoxy resin. Based on the above studies, it is clear that CB, CN, and CG all have a positive effect on improving the flame retardancy of polymers. It is known that the three carbon nanomaterials have different geometries: CB is considered to be a zero-dimension structure, CN is regarded as a one-dimension structure, and CG is a two-dimension structure; thus it is worth investigating whether they have different behaviors in flame-retarding polymers. To the best of our knowledge, there is little work on this, and it is meaningful to explore the flame-retardant behaviors of CB, CN, and CG in magnesium hydroxide–filled EVA.

In order to insightfully investigate the effect of carbon nanomaterials with different geometries on improving the flame retardancy of magnesium hydroxide–filled ethylene-vinyl acetate matrix (EM), CB, CN, and CG were chosen. In this work, the influence of these carbon materials on thermal stability, burning behavior, synergistic interaction, and mechanical properties of EVA/MH composites was also investigated.

## 2. Experimental

### 2.1. Materials

Ethylene-vinyl acetate (EVA) copolymer (Elvax-265, containing 28 wt % vinyl acetate) was supplied by DuPont Company (Wilmington, DE, USA). Magnesium hydroxide (MAGNIFIN H-5, with a content of MH more than 99.8%) was provided by Albemarle Corporation (Charlotte, NC, USA). Carbon black (particle diameter of 17 nm, purity > 99%) was purchased from Linzi Qishun Chemical Co. (Zibo, China). Carbon nanotube (10–30 nm, purity > 99%) was supplied by Chengdu Organic Chemicals Co. Ltd., Chinese Academy of Sciences (Chengdu, China). Graphite (325 mesh, purity > 99%) was purchased from Nanjing JCNANO Technology Co., Ltd. (Nanjing, China). AR grade sulfuric acid, potassium permanganate, hydrogen peroxide, and barium chloride were supplied by Tianjin Kemiou Chemical Reagent Co., Ltd. (Tianjin, China).

### 2.2. Preparation of Graphene

Graphene was prepared by the oxidation of natural graphite powder according to Hummers’ method [26,27,28]. First, a beaker with 70 mL H_2_SO_4_ liquid was put in an ice bath and 3.0 g graphite powder was added to the liquid under stirring. Successively, 9.0 g KMnO_4_ was slowly added to the suspension solution with the temperature lower than 20 °C for 2 h. Then, the suspension solution was transferred to an oil bath at 40 °C, stirring continuously for 0.5 h. A further 150 mL water was added into the solution and heated to 90 °C. The solution was stirred for 15 min and an additional 500 mL water was added. After that, 15 mL H_2_O_2_ was slowly added to the solution until the color changed from dark brown to yellow. Finally, the solution was filtered and washed with 1/10 HCl aqueous solution until sulfate radical was not detected by barium chloride solution. The filter cake was dried in the air and screened by 325 standard mesh.

### 2.3. Preparation of EVA Composites

All EVA compounds with carbon fillers of variable sizes and geometries were processed under identical conditions using a micro compounder (MC-15, Xplore Instruments BV, Sittard, the Netherlands). Processing of the composites was carried out at a temperature of 180 °C with a screw speed of 50 rpm and time of 15 min. The samples for the flame test and mechanical property test were obtained by using a hot press. All the samples were dried in a vacuum oven at 80 °C for 2 h before processing. The formulations are given in Table 1, and the corresponding materials were named magnesium hydroxide–filled EVA (EM), magnesium hydroxide–filled EVA/carbon black (EMCB), magnesium hydroxide–filled EVA/carbon nanotube (EMCN), and magnesium hydroxide–filled EVA/grapheme (EMCG).

### 2.4. Characterization and Measurement

Thermogravimetric analysis (TGA) was performed with a TA thermogravimetric analyzer (Q50, New Castle, PA, USA) from 50 to 600 °C, with a heating rate of 10 °C/min in nitrogen atmosphere. Limiting oxygen index (LOI) was obtained using an oxygen index meter (FTT, East Grinstead, UK) according to American Society for Testing and Materials (ASTM) D2863-77 standard. The size of the samples was 130 × 6.5 × 3 mm^3^.

The vertical burning test was determined with the UL-94 vertical flame chamber (FTT, East Grinstead, UK) according to ASTM D3801 standard. The size of the samples was 130 × 13 × 3 mm^3^.

**Figure 1:**
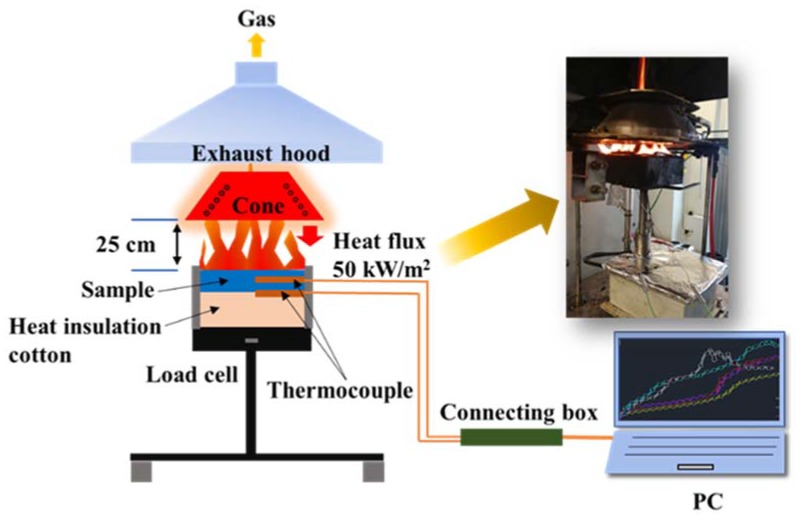
Schematic of experimental setup used for temperature measurements.

The fire behavior of the samples was determined on a cone calorimeter (FTT, East Grinstead, UK) according to the ISO5660 standard, under a heat flux of 50 kW/m^2^ with a sample size of 100 × 100 × 3 mm^3^. The temperature detection was carried out during the cone calorimeter test (CCT) with K-type thermocouple. The sample temperatures at the middle and bottom layers during combustion were monitored. The middle and bottom thermocouples were fixed by the premade hole and sample holder, respectively. Figure 1 shows the experimental setup for cone calorimeter tests and temperature measurements.

The dispersion state of different fillers in EVA matrix were examined with a scanning electron microscope (Helios NanoLab 600i, FEI, Portland, OR, USA). The samples were fractured in liquid nitrogen, and the fracture surfaces were coated with gold before SEM observation.

Tensile testing was performed on a universal electromechanical testing machine (INSTRON 3384, Norwood, MA, USA) according to ASTM D 638 standard at a test speed of 50 mm/min.

Dynamic mechanical properties were measured by a DMA Q800 (TA Instruments, New Castle, PA, USA). The dynamic storage modulus was determined at a frequency of 1 HZ and a heating rate of 3 °C/min between −50 and 50 °C.

## 3. Results and Discussion

### 3.1. Thermal Stability

**Figure 2:**
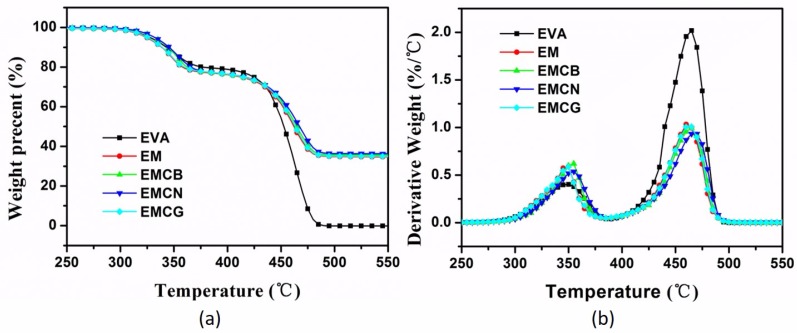
(**a**) TGA and (**b**) DTG curves of pure EVA and its composites at a heating rate of 10 °C/min in nitrogen.

The thermal stability of EVA and its composites with MH and carbon materials was investigated by TGA in nitrogen atmosphere. The TGA and derivative thermogravimetry (DTG) curves of neat EVA and its nanocomposites are plotted in Figure 2, and the corresponding data are listed in Table 2. It was observed that all samples exhibited a distinct two-step decomposition process. The first stage was due to the loss of acetic acid in EVA and the dehydration of MH from 300 to 400 °C. The second stage involved volatilization of the residual polymer and degradation of ethylene-based chains [29,30]. As shown in Figure 2, the pure EVA maximum weight-loss temperature (*T*_max1_ and *T*_max2_) for the two decomposition steps was 348 and 428 °C, respectively. Compared with the pure EVA and EM, EM with carbon nanomaterials had a higher initial decomposition temperature (*T*_−5%_) and *T*_max1_ because carbon nanomaterials are highly thermal stable; besides, all char yields had a slight increase, which was higher than 34.5 wt % of EM, indicating that carbon nanomaterials were beneficial in promoting char formation. It should be noted that the thermal stability of EMCB, EMCN, and EMCG EMCN was improved, because the MH with numerous interconnection carbon materials acted as a barrier, hindering the transport of degradation products and promoting the formation of stable charred layers [31].

### 3.2. LOI and UL-94

**Figure 3:**
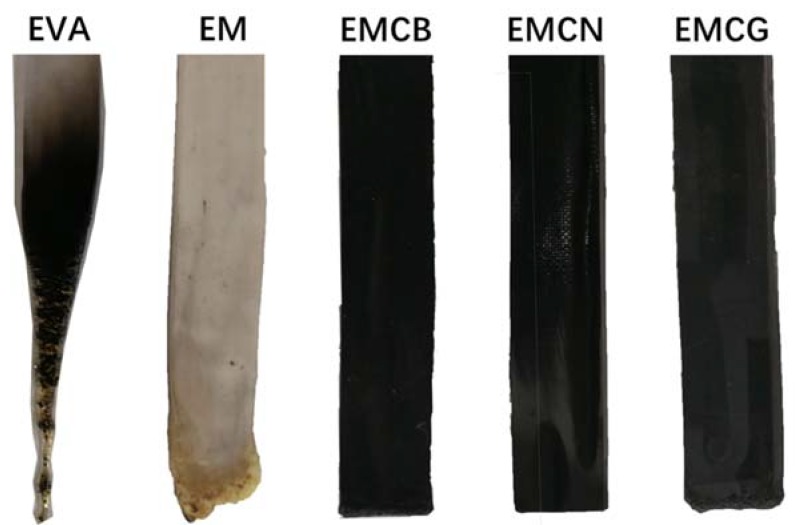
Digital photos of EVA composites after UL-94 tests.

**Figure 4:**
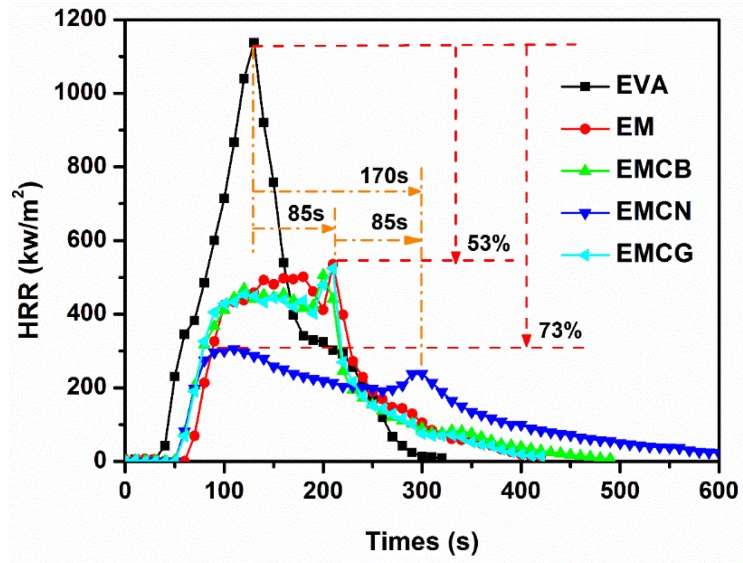
Heat release rate curves of EVA and its composites measured by a cone calorimeter at an external radiant flux of 50 kW/m^2^.

The effect of MH and different carbon materials on the flammability of EVA was studied by limiting oxygen index (LOI) and UL-94 vertical burning test (Table 3). Digital photos of EVA composites after UL-94 test are displayed in Figure 3. It can be seen in Table 3 that the LOI value of pure EVA was only 18.5% and it failed to pass UL-94 rating. When 50 wt % MH was added, the LOI value of EM increased to 25.8% and EM got a UL-94 V-1 rating. After 1 wt % carbon nanomaterials was added to the EM system, all of the EM composites with carbon materials passed UL-94 V-0 rating, and the LOI values of EMCB, EMCN, and EMCG further increased to 28.2%, 33.3%, and 27.6%, respectively. It can be seen that the LOI value of EMCN was the highest among EM with carbon nanomaterials and increased by 80% in comparison to pure EVA and 29% in comparison to EM. Figure 4 shows that EVA had significant melting and dripping, and EM had a little dripping and presented better flame-retardant performance than EVA, while the EM with carbon nanomaterials kept the original shape and had no dripping during UL-94 testing, indicating that the addition of carbon nanomaterials might enhance the melt viscosity of the matrix. All in all, it was concluded that all three carbon nanomaterials were beneficial in improving the flame-retardant efficiency of MH in EVA matrix, which was in the order of CN > CB > CG.

### 3.3. Cone Calorimeter Test

**Figure 5:**
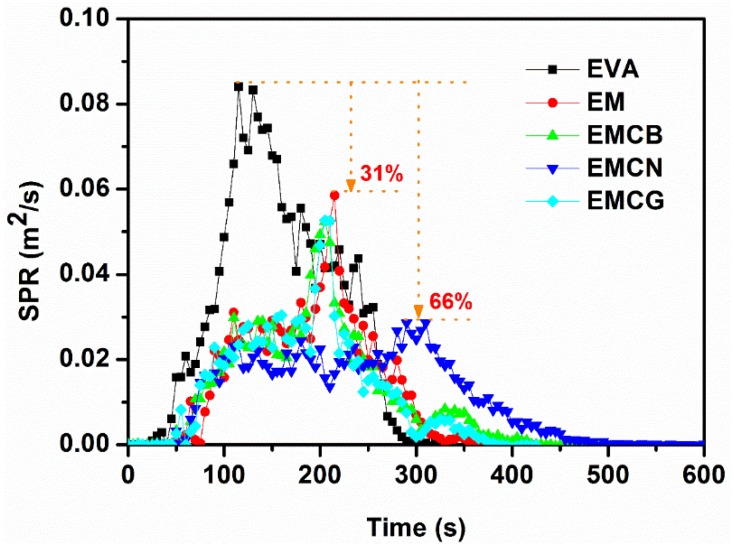
Smoke production rate of EVA and its composites measured by cone calorimeter at an external radiant flux of 50 kW/m^2^.

Cone calorimetry is an effective method to study the combustion behavior of polymers [32,33], and was adopted to assess the fire performance of EVA and EVA-based composites. The related combustion data, including heat release rate (HRR), total heat release (THR), smoke production rate (SPR), total smoke production (TSP), and residue, are summarized in Table 4, and the curves of HRR and SPR versus time for EVA and its composites are presented in Figure 4 and Figure 5.

From Figure 4, it can be seen that the pure EVA was easily flammable after ignition, exhibiting a sharp peak in the HRR curve at 125 s, with peak value at 1139 kW/m^2^. When 50 wt % MH was added to EVA, the PHRR of EM decreased remarkably from 1139 kW/m^2^ of EVA to 536 kW/m^2^, corresponding to a 53% reduction compared to pure EVA. After carbon nanomaterials were added into the EM system, the EM with carbon samples burned more slowly than pure EVA and EM. Furthermore, compared with pure EVA, EMCB, EMCN, and EMCG composites, the peak of HRR curves decreased from 1139 to 506, 308, and 564 kW/m^2^, respectively. The PHRR of EMCN was 305 kW/m^2^, corresponding to a 73% reduction compared to pure EVA and 20% reduction compared to EM. It can also be observed from Figure 4 that pure EVA burned very fast after ignition. The second sharp HRR curve appeared near 125 s. For the sample with MH, its second HRR was delayed to 210 s. With the addition of 1 wt % CB and CG in EM, the time of appearance of the second HRR remained nearly unchanged. However, in the EMCN sample with 1.0 wt % CN, its second HRR was lower than the first peak and was prolonged to 295 s. The HRR of all of flame-retardant samples with carbon materials showed flat progress during burning, which indicates that the carbon materials facilitated the formation of a protective char layer. As a result, the combustion was suppressed. Lower HRR value is crucial for saving lives and assets during a fire. Compared with CB and CG, CN showed better flame-retardant efficiency with MH in EVA matrix.

The smoke performance of flame-retardant material is a very important parameter [34]. The SPR values of EVA and EVA-based composites are illustrated in Figure 5. It was seen that EVA had a high peak SPR value of 0.084 m^2^/s. With the addition of 50 wt % MH, the peak SPR value of EM decreased dramatically to 0.058 m^2^/s, corresponding to a 31% reduction compared to pure EVA, while in the presence of carbon nanomaterials, the peak SPR values of EMCB, EMCN, and EMCG composites further decreased from 0.058 m^2^/s of EM to 0.053, 0.029, and 0.052 m^2^/s, respectively. The results indicate that CN gave rise to the best suppression effect on the smoke production of EM compared with CB and CG.

### 3.4. Flame-Retardant Mechanism

**Figure 6:**
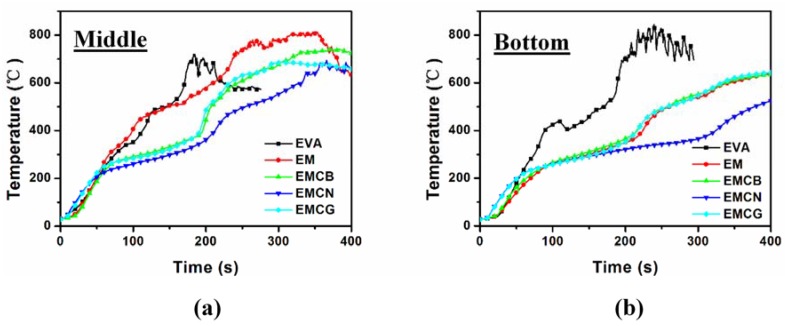
Temperature of specimens versus time for pure EVA and its nanocomposites: (**a**) middle temperature of specimen, and (**b**) bottom temperature of specimen.

**Figure 7:**
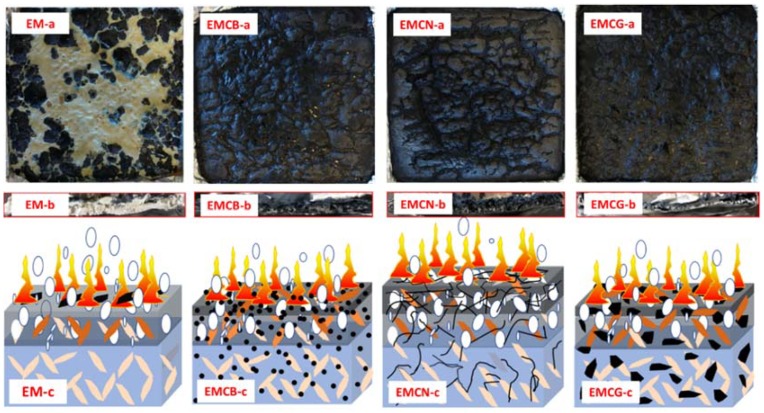
Cross-section pictures of residue obtained by interrupted irradiation and combustion diagram under cone calorimeter at 125 s. (**a**) upper surface; (**b**) cross-section; (**c**) combustion schematic diagram.

According to the above results, it was confirmed that the flame-retardant efficiency of CB, CN, and CG was very different, especially for CN, and that CN showed the best synergistic effect with MH on flame-retarding EVA matrix. In order to investigate the flame-retardancy mechanism, an online temperature monitor with thermocouples placed in the middle and bottom of the specimen was adopted to monitor the real-time temperature during combustion, and the curves of temperature versus time for EVA and EVA-based composites are recorded in Figure 6. Interrupted combustion tests were also performed, together with a visual analysis of cross-sections of fire residues. According to the time of PHRR of EVA, interrupted combustion happened 125 s after the start of burning. Pictures of residues and cross-section pictures of residue obtained by interrupted combustion are shown in Figure 7.

As shown in Figure 6a, the middle of the thermocouple detected the approximately similar increase in temperature for EVA and the EM composite. The EMCB and EMCG composites showed similar and nonsignificant differences during combustion. It is worth noticing that the temperature curve of EMCN was lower than other curves after approximately 50 s of burning. This was because the barrier layer had not formed on top of the specimen, and it did not effectively block the heat transfer at the initial combustion stage. In Figure 6b, it can be seen that the increased temperature rate of EVA was higher than that of EVA composites after approximately 50 s; EM, EMCB, and EMCG temperature curves coincided during burning; and the EMCN temperature curve appeared lower after approximately 125 s. With the development of the combustion process, a heat barrier was formed by MH and carbon materials. Consequently, the char layer had a stronger and stronger effect on combustion and delayed the temperature increase in the deeper parts of the specimen. Compared to EM, EMCB, and EMCG, the char of EMCN presented a better barrier effect. Based on the above cone calorimeter test results of residue, the better barrier effect of the residue mass was stronger than other samples.

As seen in Figure 7a, the EM composite cross-sections show the presence of voids originated by fuel bubbles in the whole thickness of the polymer, and a few plate-like chars appeared on the surface and did not cover the sample entirely. This indicates that the addition of MH into EVA matrix did not generate the obvious flame-retardant layer to prevent heat transport from flame into the materials. In Figure 7b–d, a similar fuel bubble feature can be observed in the cross-section of the composites. However, after adding carbon materials at 1.0 wt % concentration to EVA and MH system, a layer of char appeared on top of the samples, indicating that the addition of carbon nanomaterials obviously increased the char residue of EM composite.

**Figure 8:**
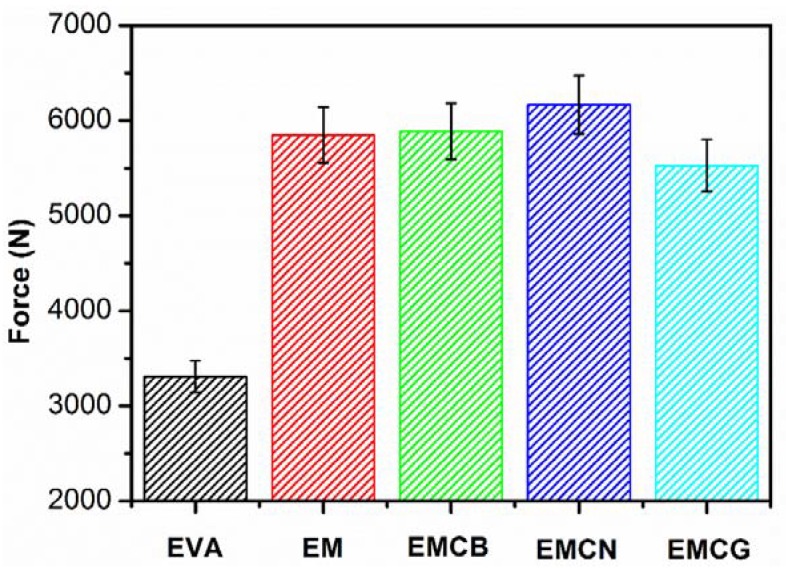
Force of EVA with MH and different carbon materials after mixing for 15 min at 180 °C.

Takashi et al. demonstrated that the formation of a network structure of nanoparticles within a polymer matrix can significantly reduce nanocomposite flammability, and that viscoelastic properties could be utilized to predict that reduction [31,35,36]. The torque of the melting polymer is associated with melt viscosity and is extensively used to characterize the evolution of the polymer mixing process [37,38]. Figure 8 shows the applied torque of EVA with different fillers after mixing for 15 min at 180 °C an average of nine times. The torque force value of pure EVA was 3200 N; after the addition of MH, the value increased to 5850 N. This indicates that the filler of MH can remarkably increase the viscosity of EM composite. Three carbon materials were added into the EM system, and the value of force presents different results. Compared to EM, the value of EMCN slightly increased, EMCN obviously increased, and EMCG reduced. Because CB has much smaller particles than MH, it cannot obviously influence the viscosity of EM composite. The reduction in EMCG was due to the layered structure of graphene, which had a lubrication effect in the processing of EVA. The increase of EMCN was attributed to the presence of long carbon nanotubes and the formation of network structures with polymer matrix in the nanocomposites. It is worth noticing that the EMCN composite presented better flame-retardant performance than other composites due to the formed network [31,36]; the high melt viscosity reduced the bubble rise rate and bubble growth rate of volatile degradation products. This EMCN structure greatly increased the thickness and integrity of the charred layer, acted as a heat insulator, limited the emission of volatile thermal degradation products, and thus improved the thermal oxidative stability [38].

From the above discussion, we concluded that the flame-retardant mechanism of EMC composites was mainly in the condensed phase. The burning process and flame-retardant mechanism can be described as follows [36]. First, the materials were heated to a temperature at which the polymer was melting and bubbles began to form, where thermal degradation occurs, and they grew with the supply of more degradation products by diffusion from the surrounding molten plastic. When the temperature of materials reached ignition temperature, it began to burn. At the same time, during the heating process, MH was decomposed into magnesium oxide and water, and the released water vapor isolated the flame and diluted the combustible gas in the gas phase. Meanwhile, the accumulated carbon material formed a layer of char by working with magnesium oxide, which was a thermal barrier. This charred layer prevented heat transport of degradation products between the molten polymer and the surface.

### 3.5. Mechanical Properties

In this part, the influence of different fillers on the mechanical properties of EVA composites was investigated. The detailed data are given in Table 5. As can be seen from Table 5, both the tensile strength and elongation at break of EM and EMC materials significantly decreased compared to neat EVA. Compared with EM, the tensile strength and elongation at break of EMCB and EMCG were nearly unchanged, while for EMCN, both tensile strength and elongation at break slightly decreased compared with EM. This indicated that it had not been adversely affected in mechanical properties when few carbon materials were added into the EM system.

**Figure 9:**
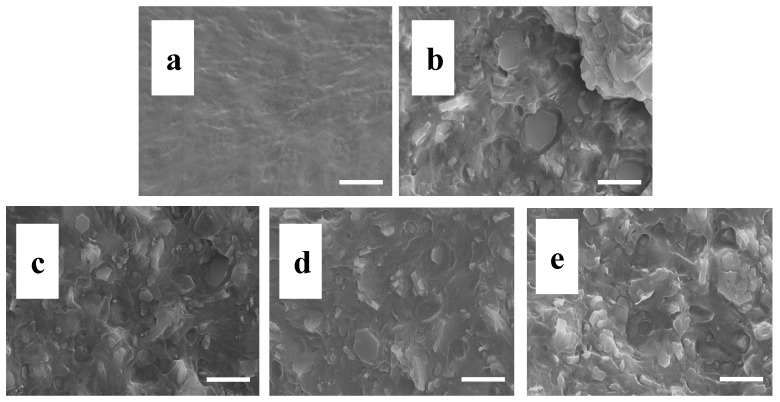
SEM micrographs of the brittle-fractured surface of EVA and its composites: (**a**) pure EVA, (**b**) EM, (**c**) EMCB, (**d**) EMCN, (**e**) EMCG. (Scale bar = 2 μm).

To understand the dispersion levels of different filler particles in EVA, SEM was used to characterize the freeze-fractured surface microstructure of EVA and its composites. Figure 9 shows SEM micrographs of the freeze-fractured surfaces of EM, EMCB, EMCN, and EMCG. According to the previous studies, the fracture roughness of the polymer nanocomposites reflects the dispersion level and interfacial interaction to some degree [36,39]. It can be observed in Figure 9a that the neat EVA displayed a smooth fracture surface. However, after the MH was mixed with the EVA, the MH particles imaged on the fracture surface of the EVA nanocomposites can be clearly seen in Figure 1b and Figure 9c–e. In addition, it was observed that the surface of EMCN was smoother than other flame-retardant composites, which can explain why the tensile stress value of EMN was lower than other composites.

**Figure 10:**
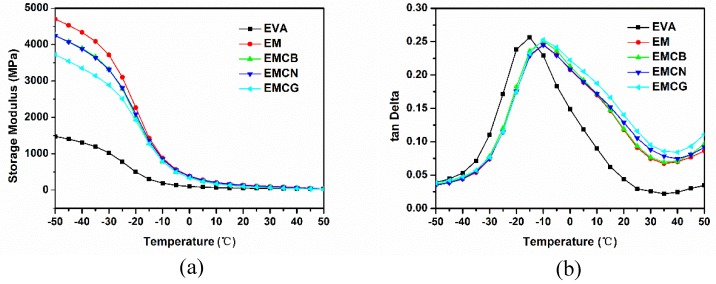
Temperature dependence of (**a**) storage modulus and (**b**) tan δ of pure EVA and its composites.

To investigate the influence of MH and carbon materials on the dynamic mechanical behavior of EVA composites, the storage modulus and tan δ as the function of temperature were used, shown in Figure 10. From Figure 10a, we can conclude there were no obvious differences in modulus for three samples above 0 °C, while below 0 °C, the storage modulus of EVA composites was higher than that of neat EVA, indicating that EVA composites were more rigid than EVA. This is because the rigid filler imparted stiffness behavior to the filler EVA composites [6]. From Figure 10b, it can be seen that the glass transition temperatures (defined as the temperature at the peak value of tan δ) of EVA composites were all higher than that of pure EVA, which was ascribed to the rigid fillers limiting the mobility of the polymer chains [30].

## 4. Conclusions

EM with different geometries of carbon materials in EVA matrix was prepared by melt-blending. The TGA results show that EMCN had the best thermal stability. The flame-retardant properties show that the LOI values of EMCB, EMCN, and EMCG were 28.2%, 33.3%, and 27.6%, respectively. Meanwhile, all the EM materials with carbon materials passed UL-94 V-0 rating. The combustion behavior results indicate that carbon materials acting as flame-retardant synergistic agents of MH highly improved the flame retardancy of EVA composites, and the synergistic effects were in the order of one-dimensional CN > zero-dimensional CB > two-dimensional CG. The torque results demonstrate that carbon nanotubes with MH and CN formed network structures with polymer matrix in the nanocomposites. The flame-retardant mechanism of composites was mainly in the condensed phase. The incorporation of carbon materials did not have a negative effect on the tensile performance of EM composites, and there were also no obvious differences in modulus for all samples above 0 °C, while below 0 °C, the storage modulus of EVA composites was higher than that of pure EVA. It was demonstrated that the geometry of carbon material with MH in EVA matrix plays a key role in structure-property relationships.

## Figures and Tables

**Table 1:** Formulations of ethylene-vinyl acetate (EVA) composites.

Sample	EVA (wt %)	MH (wt %)	CB (wt %)	CN (wt %)	CG (wt %)
EVA	100	0	0	0	0
EM	50	50	0	0	0
EMCB	50	49	1	0	0
EMCN	50	49	0	1	0
EMCG	50	49	0	0	1

MH, magnesium hydroxide; CB, nanocarbon black; CN, carbon nanotube; CG, graphene; EM, magnesium hydroxide–filled EVA; EMCB, magnesium hydroxide–filled EVA/carbon black; EMCN, magnesium hydroxide–filled EVA/carbon nanotube; EMCG, magnesium hydroxide–filled EVA/grapheme.

**Table 2:** TGA and DTG data of pure EVA and its composites in nitrogen.

Sample	*T*_−5%_^a^ (°C)	*T*_−50%_^b^ (°C)	*T*_max1_^c^ (°C)	*T*_max2_^d^ (°C)	Char ^e^ (%)
EVA	327	452	348	466	0
EM	324	462	348	461	34.5
EMCB	331	464	353	464	35.5
EMCN	332	467	354	468	36.1
EMCG	325	463	349	463	35.0

^a^ Temperature at 5 wt % weight loss. ^b^ Temperature at 50 wt % weight loss. ^c^ Temperature at first maximum mass loss rate. ^d^ Temperature at second maximum mass loss rate. ^e^ Residue at 600 °C.

**Table 3:** Limiting oxygen index (LOI) and UL-94 results.

Samples	LOI (%)	UL-94				
		*t*_1_ (s)	*t*_2_ (s)	Dripping	Igniting the Cotton	Rating
EVA	18.5 ± 0.2	/	/	Yes	Yes	Fail
EM	25.8 ± 0.2	2	9	Yes	No	V-1
EMCB	28.2 ± 0.2	1	2	No	No	V-0
EMCN	33.3 ± 0.2	1	1	No	No	V-0
EMCG	27.6 ± 0.2	1	3	No	No	V-0

**Table 4:** Combustion parameters obtained from cone calorimetry test.

Sample	*t*_ign_ (s)	PHRR (kW/m^2^)	THR (MJ/m^2^)	SPR (m^2^/s)	TSP (m^2^/kg)	esidue (wt %)
EVA	36 ± 2	1139 ± 50	110 ± 5	0.084 ± 0.004	10.0 ± 0.5	0.0
EM	66 ± 2	536 ± 20	85 ± 5	0.058 ± 0.002	5.9 ± 0.2	40.4 ± 1.0
EMCB	55 ± 1	506 ± 20	84 ± 5	0.052 ± 0.002	5.3 ± 0.2	41.6 ± 1.5
EMCN	50 ± 2	308 ± 15	83 ± 4	0.029 ± 0.001	6.4 ± 0.3	48.7 ± 2.0
EMCG	54 ± 1	564 ± 20	82 ± 4	0.053 ± 0.002	5.5 ± 0.2	42.9 ± 1.0

**Table 5:** Mechanical properties of pure EVA and its nanocomposites.

Sample	Tensile Strength (MPa)	Elongation at Break (%)
EVA	23.9 ± 0.5	1286 ± 50
EM	10.5 ± 0.3	753 ± 30
EMCB	10.6 ± 0.2	758 ± 25
EMCN	9.8 ± 0.2	612 ± 25
EMCG	10.7 ± 0.3	634 ± 25
